# Switching of Hormone Therapies in Breast Cancer Women

**DOI:** 10.1055/s-0040-1719149

**Published:** 2021-01-19

**Authors:** Luana Moreira de Medeiros, Rebeca Stahlschmidt, Amanda Canato Ferracini, Cinthia Madeira de Souza, Cassia Raquel Teatin Juliato, Priscila Gava Mazzola

**Affiliations:** 1 Faculty of Pharmaceutical Sciences, Universidade de Campinas (Unicamp), Campinas, SP, Brazil; 2 Graduate Program in Medical Sciences, Faculty of Medical Sciences, Universidade de Campinas (Unicamp), Campinas, SP, Brazil; 3 Department of Obstetrics and Gynecology, Faculty of Medical Sciences, Universidade de Campinas (Unicamp), Campinas, SP, Brazil

**Keywords:** breast cancer, tamoxifen, hormone therapy, aromatase inhibitor, anastrozole, câncer de mama, tamoxifeno, terapia hormonal, inibidor da aromatase, anastrozol

## Abstract

**Objective** The objective of the present study was to analyze the reasons that led to hormone therapies (HTs) regimen changes in women with breast cancer.

**Methods** This was a retrospective cross-sectional study from a single-institution Brazilian cancer center with patient records diagnosed with breast cancer between January 2012 and January 2017.

**Results** From 1,555 women who were in treatment with HT, 213 (13.7%) women had HT switched, either tamoxifen to anastrozole or vice-versa. Most women included in the present study who switched HT were > 50 years old, postmenopausal, Caucasian, and had at least one comorbidity. From the group with therapy change, ‘disease progression’ was reason of change in 124 (58.2%) cases, and in 65 (30.5%) patients, ‘presence of side effects’ was the reason. From those women who suffered with side effects, 24 (36.9%) had comorbidities.

**Conclusion** The present study demonstrated a low rate of HT switch of tamoxifen to anastrozole. Among the reasons for changing therapy, the most common was disease progression, which includes cancer recurrence, metastasis or increased tumor. Side effects were second; furthermore, age and comorbidities are risk factors for side effects.

## Resumo

**Objetivo** O objetivo do presente estudo foi analisar os motivos que levaram às mudanças no esquema hormonioterápico (HT) em mulheres com câncer de mama.

**Métodos** Estudo transversal retrospectivo realizado no Hospital da Mulher de Campinas e consequente pesquisa de prontuários de mulheres diagnosticados com câncer de mama entre janeiro de 2012 e janeiro de 2017.

**Resultados** De 1.555 mulheres em tratamento com HT, 213 (13,7%) mulheres tiveram HT alterado, tamoxifeno para anastrozol ou vice-versa. A maioria das mulheres incluídas no presente estudo que tiveram mudança de HT tinha > 50 anos, estava na pós-menopausa, era caucasiana e tinha pelo menos uma comorbidade. Os principais motivos de troca de HT foram devido a 'progressão da doença', ocorrendo em 124 (58,2%) casos e a 'presença de efeitos colaterais' ( *n =* 65; 30,5%). Das mulheres que sofreram efeitos colaterais, 24 (36,9%) apresentaram comorbidades.

**Conclusão** O presente estudo demonstrou uma baixa taxa na alteração de tamoxifeno para anastrozol. Entre as razões mais comuns para alterar a HT estava a progressão da doença, que inclui recorrência do câncer, metástase ou aumento do tumor. Os efeitos colaterais foram a segunda causa e, além disso, a idade e as comorbidades foram fatores de risco para efeitos colaterais.

## Introduction

Breast cancer (BC) is the most common type of gynecological cancer, and it is the highest cancer-related cause of death among them. 1 Oncological therapy includes surgery, radiotherapy and systemic therapy, as chemotherapy and hormone therapy. 2 Hormone therapy (HT), such as anastrozole and tamoxifen, is used for patients whose tumor is positive for estrogen receptor, which reaches 70% of BC cases. These tumors grow and proliferate as a result of estrogen action on its receptors. 3

According to international guidelines, HT is an extensive treatment with duration of 5 to 10 years, and adherence and adequate treatment duration is crucial to the effectiveness of the treatment. Although these classes of drugs are well tolerated, the drug-related side effects are one of the most important causes for non-adherence or treatment discontinuation. 4 5 6

Although there are options to investigate symptoms related to side effects, for which medication switch is one of the strategies, causes of these changes and its relationship with patient outcomes in therapy are still poorly studied. Because HTs are commonly dispensed through pharmacy channels, their significant potential adverse events, adherence, and high patient self-management requirements suggest the clinical pharmacist to drug therapy management. 7 In the present study, we aimed to access the reasons for changes in the HT regimen in patients treating BC. Then, we evaluate its frequency and its relationship with disease progression, side effects and comorbidities.

## Methods

This was a cross-sectional study. Data was collected from computed records from January 2012 to January 2017, at the Hospital da Mulher of the Universidade de Campinas (UNICAMP, in the Portuguese acronym), Brazil, a public hospital specialized in women's health, being a referral center in the region for the treatment of women with BC. The inclusion criteria were medical records from: women > 18 years old with a diagnosis of BC (any stage of the disease), treated with tamoxifen or anastrozole, accompanied at the hospital, and who had change of treatment with HT out of the suggested period of the international protocol, either from tamoxifen to anastrozole or vice-versa. 8 The exclusion criterion was medical records that did not describe the reason for change in therapy. The present study was approved by the Research Ethics Committee (CAAE: 54977116.0.0000.5404). All procedures were performed according to the 1946 Helsinki Declaration

The patients (or a legal responsible) authorized access to their computed record containing information about pick up of HT. Changes in the dispensation pattern was the trigger to analyze medical records. Researchers did not evaluate or follow the prescribed change in the treatment. Other data such as age, ethnicity, menopausal state, onset of treatment, date of treatment change, reason(s) for change, presence of comorbidities, which change of treatment occurred, and date of death, if it occurred, were obtained from the clinical files.

Disease progression was considered cancer recurrence, at least 20% increase in tumor or more, new lesions and metastasis to other locations. Overall survival (OS) was calculated as the number of months from cancer diagnosis to death or the last follow-up. 9

Side effects were categorized according to the Common Terminology Criteria for Adverse Events (CTCAE) Version 5.0: cardiac disorders (ischemic events), eye disorders (cataract, glaucoma and blurred vision), gastrointestinal disorders (nausea, vomit, diarrhea, esophageal pain), general disorders and administration site conditions (fatigue), musculoskeletal and connective tissue disorders (arthralgia, chest wall pain, pain in extremity, myalgia), nervous system disorders (stroke, headache, peripheral motor neuropathy), reproductive system and breast disorders (vaginal hemorrhage, vaginal discharge), skin and subcutaneous tissue disorders (pruritus, rash maculopapular, dry skin and hyperpigmentation), and vascular disorders (thromboembolic events, hot flashes, phlebitis). 10 In cases of side effects not categorized in this reference (i.e.: criterion of classification is not specific to tamoxifen or anastrozole), these were classified as “without classification.”

After data collection, statistical analysis was performed. The chi-squared test was used for categorical variables, and the Mann-Whitney test for numeric variables. To describe the sample profile, data was organized in tables to obtain absolute frequency and percentage. Associations were analyzed among (1) comorbidities with side effects and treatment duration; (2) side effects; and (3) disease progression. When two side effects occurred for the same patient and both were decisive factors for the change in therapy, they were amounted as two symptoms. The Cox hazards model was used to identify variables that predict OS analysis. Calculations were performed using hazard ratio (HR) values and 95% confidence intervals (95% CIs). Differences were significant when *p* < 0.05.

## Results

From 1,555 women treated with HT, 213 (13.7%) underwent a HT change. Most of the women included in the present study who switched HT were > 50 years old, the average age was 61.4 ± 13.7, postmenopausal, Caucasian, and had at least one comorbidity ( Table 1 ). Forty-nine women died.

**Table 1:** Demographic characteristics of the study population

Characteristic	*n*	(%)
Age (years old)		
20–49	37	17.4
≥ 50	176	82.6
Ethnicity		
Caucasian	185	86.9
Non-caucasian	28	13.1
Menopause status		
Premenopausal	49	23
Postmenopausal	154	72.3
Ignored	10	4.7
Comorbidity *		
Yes	137	64.3
No	76	35.7

* Comorbidity is all diseases besides breast cancer.

Most of the patients included in the present study, 208 (97.7%), changed tamoxifen to anastrozole therapy, and only 5 (2.3%) women changed anastrozole to tamoxifen. The major reason for treatment changes was disease progression like cancer recurrence, increased tumor or metastasis, 124 (58.2%), followed by side effects, 65 (30.5%).

The category “confirmation of menopause” aimed to confirm menopausal status either because the patient reached menopause naturally or caused by ovarian ablation, which is one of the reasons for changing therapy from tamoxifen to anastrozole.

The category “risk” included patients who presented comorbidities that could increase the risk of some side effects, such as cardiovascular problems, endometrial thickening, or osteoporosis. The category “others” included women who had changes in therapy, either because they reached the total time of the treatment protocol, had drug interaction, advanced age or allergies ( Table 2 ). The main side effects are described in Table 3 , the most common being vascular disorders, without classification, followed by skin and cutaneous disorders.

**Table 2:** Reasons for change in hormone therapy

Modification	*n*	%
Reason of change		
Disease progression	124	58.2
Side effect	65	30.5
Risk *	11	5.2
Non-adherence	5	2.3
Confirmation of menopause	4	1.9
Others	4	1.9
Total	213	100

* Risk of patient sufferiing side effects due to her comorbidities.

**Table 3:** Characteristics and frequency of side effects, according to the Common Terminology Criteria of Adverse Events

Side effects	*n*	(%) *
Vascular disorders	19	29.2
Without classification **	14	21.5
Skin and subcutaneous tissue disorders	6	9.2
Musculoskeletal and connective tissue disorders	6	9.2
Gastrointestinal disorders	6	9.2
Nervous system disorders	4	6.2
Reproductive system and breast disorders	4	6.2
Eye disorders	3	4.6
General disorders and administration site conditions	3	4.6
Cardiac disorders	2	3.1

* Total to calculate percentage considered 65 patients, since 2 patients had two different types of side effects decisive to change their therapy.

** Side effects not classified by Common Terminology Criteria of Adverse Events (CTCAE) version 5.0.

The correlation among disease progression, side effects and treatment time was analyzed. Patients who suffered disease progression had an average of duration of treatment until the switch of therapy of 20.5 ± 13.3 months, and patients who presented side effects had an average duration of treatment until the switch of therapy of 12.4 ± 10.9 months, with *p-value* = 0.0030. Patients who presented side effects were also older (65.4 ± 13.1 years old) than women who did not present side effects (59.6 ± 13.7 years old), with *p-value* = 0.0001.

The relationship between comorbidities and side effects also was evaluated. From 65 women, 48 (73.8%) who presented side effects had at least one comorbidity, indicating that comorbidity might be a risk factor for side effects ( *p* = 0.0544). Twenty-four out of 65 (36.9%) women had at least 1 side effect-related comorbidity. Thus, a correlation between comorbidity related to its side effect and the adverse event was determined ( *p* = 0.0002).

Women who had side effects were older and had less treatment time ( *p* = 0.0030). Thus, their age can be a risk factor for side effects and the side effect itself hinders the women to proceed with the treatment even if it had been effective. In this group, statistical analysis also demonstrated that it took at least 12 months of treatment to occur a side effect was incompatible with continuing with the therapy. Furthermore, women who switched HT and presented disease progression had more risk of death than women who had side (HR: 4.112; 95% CI 1.732–9.761).

## Discussion

The present study showed that 13 in 100 women switched their HT for BC treatment. These changes were related to disease progression, comorbidities and side effects. A similar study performed in Italy, which included 939 women, found that ∼ 7.8% had switched from tamoxifen to aromatase inhibitor, 11 results lower than those of our study. Most of the therapy switches from tamoxifen to anastrozole were due to hospital protocol. Although disease progression was found as the most common reason for the switching of HT, it was not related to therapeutic ineffectiveness, since BC can progress because of other factors, such as tumor aggressiveness, stage of the disease in which the treatment was initiated, resistance to treatment, growth factors supplied to cells around the tumor, among others. 12 13

Besides these factors, there are some comorbidities that are risk factors for cancer progression like diabetes mellitus, dyslipidemia and smoking, and those comorbidities were found in some women who suffered disease progression. 14 15 16 Data of our study also points out that patients had an average of 20 months until progression of the disease. A study that evaluated hormone therapy found a progression free survival (PFS) of 8.2 to 13.8 months with anastrozole treatment, and another study had an average of 15.9 months to the PFS event, also with anastrozole treatment. 17 18

Low/medium adherence was associated with systemic side effects and higher stages of disease. 19 The cases of side effect as reason for treatment switch affected more than half of the women who had comorbidities related to the adverse event: hypertension, dyslipidemia, diabetes mellitus, smoking, and hypothyroidism, which are risk factors to vascular disorders. 20 21 22 Diabetes mellitus, besides inducing predisposition to ischemic events, also increases the risk of ophthalmic disorders like glaucoma and cataract, peripheral pain, gastrointestinal symptoms such as nausea and vomit, and hepatic steatosis. 23 24 25 26 Smoking causes predisposition for cutaneous, gastrointestinal and gynecological complications such as endometrial thickening. 27 28 29 There are evidences of therapy management in a collaborative group, improving patient's adherence, reducing adverse events and reducing costs of healthcare system. 30 Include a pharmacist in team-based to improve patient's healthcare, specialty in oncology, have shown positive results. 7 8 9 10 11 12 13 14 15 16 17 18 19 20 21 22 23 24 25 26 27 28 29 30 31

Correlation between side effects and comorbidities analysis showed that a third of patients who suffered side effects also had comorbidities that predisposed to those side effects. The number of patients presenting side effects could have been higher considering the patients who were early identified with comorbidities that increase the risk of side effects, and had their treatment switched preventing severe adverse events.

The retrospective design of the present study is a limitation. The routine professional practice, such as HT management by a pharmacist, is essential to evaluate the causes of these changes and its relationship with patient outcomes. In this context, prospective and clinical studies should be conducted to analyze other variables.

The present study evidences better understanding and knowledge of changes in HT regimen and that healthcare professionals, including pharmacists, should be more attentive to the comorbidities of their patients and other clinical factors, since these elements can increase the risk of the occurrence of a serious adverse event that can be avoided. A cost-effectiveness study should be planned in order to understand the impact on different models of health systems.

## Conclusion

The present study demonstrated a low rate of HT switch in women with BC. Among the causes for therapy changes, disease progression, which includes cancer recurrence, metastasis or increased tumor, was the most common. Side effects were second, and age as well as comorbidities indicated risk factors of side effects.
